# Independent Associations Between Different Measures of Socioeconomic Position and Smoking Status: A Cross-Sectional Study of Adults in England

**DOI:** 10.1093/ntr/ntaa030

**Published:** 2020-02-06

**Authors:** Emma Beard, Jamie Brown, Sarah E Jackson, Robert West, Loren Kock, Sadie Boniface, Lion Shahab

**Affiliations:** 1 Research Department of Behavioural Science and Health, University College London, London, UK; 2 Institute of Alcohol Studies, London, UK; 3 Addictions Department, Institute of Psychiatry Psychology & Neuroscience, Kings College London, London, UK

## Abstract

**Introduction:**

To gain a better understanding of the complex and independent associations between different measures of socioeconomic position (SEP) and smoking in England.

**Aims and Methods:**

Between March 2013 and January 2019 data were collected from 120 496 adults aged 16+ in England taking part in the Smoking Toolkit Study. Of these, 18.04% (*n* = 21 720) were current smokers. Six indicators of SEP were measured: social grade, employment status, educational qualifications, home and car ownership and income. Models were constructed using ridge regression to assess the contribution of each measure of SEP, taking account of high collinearity.

**Results:**

The strongest predictor of smoking status was housing tenure. Those who did not own their own home had twice the odds of smoking compared with homeowners (odds ratio [OR] = 2.01). Social grade, educational qualification, and income were also good predictors. Those in social grades C1 (OR = 1.04), C2 (OR = 1.29), D (OR = 1.39), and E (OR = 1.78) had higher odds of smoking than those in social grade AB. Similarly, those with A-level/equivalent (OR = 1.15), GCSE/vocational (OR = 1.48), other/still studying (OR = 1.12), and no post-16 qualifications (OR = 1.48) had higher odds of smoking than those with university qualifications, as did those who earned in the lowest (OR = 1.23), third (OR = 1.18), and second quartiles (OR = 1.06) compared with those earning in the highest. Associations between smoking and employment (OR = 1.03) and car ownership (OR = 1.05) were much smaller.

**Conclusions:**

Of a variety of socioeconomic measures, housing tenure appears to be the strongest independent predictor of smoking in England, followed by social grade, educational qualifications, and income. Employment status and car ownership have the lowest predictive power.

**Implications:**

This study used ridge regression, a technique which takes into account high collinearity between variables, to gain a better understanding of the independent associations between different measures of SEP and smoking in England. The findings provide guidance as to which SEP measures one could use when trying to identifying individuals most at risk from smoking, with housing tenure identified as the strongest independent predictor.

## Introduction

In England, only 15% of the adult population smoke but prevalence is substantially higher among those who are socially disadvantaged.^1^ Twenty-six per cent of those working in routine and manual occupations are smokers, compared with 10.2% of those in managerial and professional occupations and 16.2% in intermediate occupations.^2^ This pattern is observed across a diverse range of socioeconomic measures including wealth, education, income, housing tenure, and car ownership.^1^ There also appears to be a cumulative effect, with the highest smoking rates among localities characterized by single-parent households, individuals who do not own a home, with little community support, no access to a car and few educational qualifications.^3^

In order to inform interventions and policies aimed at reducing inequalities in health, it is important to gain a better understanding of which socioeconomic measures are *independently* most strongly associated with smoking status. As socioeconomic position (SEP) measures are typically highly correlated, traditional statistical approaches are unable to isolate the variables producing the largest independent associations.^4^ A statistical technique that can overcome this is ridge regression, which comes from the machine learning arena.^5^ This paper applies this novel technique to assess the associations of multiple measures of SEP with smoking status.

Each measure of SEP has strengths and limitations. For example, income gives a good indicator of access to material goods and services that could influence health, but it is affected by high nonresponse rates, social desirability, fluctuations over time, retirement, and the fact that household savings are not captured. Car ownership was historically seen as a good marker of material living standards and was incorporated in several measures of area deprivation,^6^ but no longer discriminates well between socioeconomic groups^7,8^ and may be less predictive in urban areas with good public transport links. Education is easy to measure and is stable beyond adulthood, while occupation provides a link between educational measures and income. Working status can be problematic for some groups such as those who are still studying full time and those of retirement age. Finally, housing tenure—whether one owns their own home—is a good indicator of SEP as it accounts for a large proportion of outgoings from income but may be specific to the temporal and geographic context.^9^ Others have argued for the use of composite scores which integrate various individual level measures of SEP, as findings from individual measures can often result in conflicting conclusions.^10,11^ However, composite scores themselves have limitations including a reliance on complete data across measures and problems of interpretation, thereby creating difficulties for policy development, and increasing cost for survey designers.^9,10^

To our knowledge, there has been little attempt to compare and quantify the degree of independent association between a diverse range of measures relating to SEP or composite scores with smoking status. We previously used this method to identify the best SEP predictors of alcohol intake.^12^ This study found that social grade and educational attainment were the strongest SEP predictors of alcohol consumption indices in England, followed closely by housing tenure. Employment status and car ownership had the lowest predictive power.^12^ Smoking and high‐risk drinking are associated at both an individual and population level, which suggests similar findings may be likely.^13,14^ On the other hand, smoking patterns in England are more heavily driven by sociodemographic disparities than alcohol consumption.^15^ The predictive power of different measures of SEP may, therefore, differ for the two behaviors.

In summary, this paper addresses the following research questions:

1. What are the univariate associations between six individual SEP measures (ie, income, home ownership, car ownership, education, employment status, and social grade) and a composite of these with smoking status?
2. What are the independent multivariable associations between SEP measures and smoking status using ridge regression?

It is hoped that these findings will help to inform population surveys by indicating which of SEP measures are key indices of smoking behavior. This is important as logistical and financial constraints often mean it is not possible to include multiple measurements of SEP. It is also hoped the findings will help to inform policies and interventions aimed at promoting smoking cessation. For example, if lower income is highly predictive of smoking status this may lend support to fiscal policies such as tax increases (which aim to increase the cost of tobacco) as they have been shown to be most effective among more disadvantages groups,^16^ while a strong association with housing tenure would support the provision of localized anti‐smoking campaigns and neighborhood smoking cessation services in areas where social housing or renting is more common.^17^

## Materials and Methods

### Ethical Approval

The data are collected by Ipsos Mori on behalf of UCL and are anonymized before being received by UCL Approval for the study was granted by UCL Ethics Committee (ID 0498/001). Explicit verbal agreement and willingness to answer questions voluntarily is recorded electronically by Ipsos Mori. Participants are also given a printed information sheet.

### Design

Data were used from the Smoking Toolkit Study (STS; www.smokinginengland.info) between March 2013 and January 2019. The STS involves monthly cross-sectional household computer-assisted interviews of approximately 1700 adults aged 16+ and over in England.^18^ The baseline survey uses a type of random location sampling, which is a hybrid between random probability and simple quota sampling. England is first split into greater than 170 000 ‘Output Areas,’ comprising of approximately 300 households. These areas are then stratified based on A Classification of Residential Neighbourhoods (ACORN) characteristics and geographic region (http://www.caci.co.uk/acorn/). The areas are then randomly allocated to interviewers, who travel to their selected areas and conduct the electronic interviews with one member of each selected household. Strengthening The Reporting of OBservational studies in Epidemiology (STROBE) reporting guidelines are followed in this paper.^19^

### Measures

#### Dependent Variable

Smoking status was assessed by asking participants: “Which of the following best applies to you? a) I smoke cigarettes (including hand-rolled) every day; b) I smoke cigarettes (including hand-rolled), but not every day; c) I do not smoke cigarettes at all, but I do smoke tobacco of some kind (e.g. pipe or cigar); d) I have stopped smoking completely in the last year; e) I stopped smoking completely more than a year ago; f) I have never been a smoker (i.e. smoked for a year or more).” Smokers were defined as those who answered a), b) or c).

#### Independent Variables

We included all SEP measures available in the STS.

Social grade was measured using the British National Readership Survey (NRS) Social-Grade Classification Tool^20^ which categorizes individuals into AB (Higher managerial, administrative or professional), C1 (Supervisory or clerical and junior managerial, administrative or professional), C2 (Skilled manual workers), D (Semi-skilled and unskilled manual workers), and E (Casual or lowest grade workers, pensioners, and others who depend on the welfare state for their income).

Self-reported gross annual household income was measured in 15 bands: up to £4499; £4500–6499; £6500–7499; £7500–9499; £9500–11 499; £11 500–13 499; £13 500–15 499; £15 500–17 499; £17 500–24 999; £25 000–29 999; £30 000–39 999; £40 000–49 999; £50 000–74 999; £75 000–99 999; ≥ £100 000.

Educational level comprised of General Certificate of Secondary Education (GCSE)/O-level/CSE (high school sophomore); vocational qualification (high school senior); A-level or equivalent (high school senior); Bachelor degree or equivalent (university undergraduate); Masters/PhD or equivalent (university postgraduate); other; no formal qualifications (no post-16 qualifications); still studying.

Car ownership was measured by asking participants if they owned or did not own a car.

Working status was recorded in seven categories: have a paid job (full time); have a paid job (part time); self-employed; full-time student; still at school; retired; not in paid work (long-term illness, housewife or other reason).

Housing tenure was categorized as mortgage; owned outright; rented from local authority; rented from private landlord; belongs to housing association; other.

Due to violations of the assumption of linearity and in order to improve interpretation, all SEP variables except social grade were recategorized and coded so that lower SEP or greater social disadvantage reflected *higher* scores). Income was coded into four quartiles: £50 000+, £25 000–49 999, £13 500–24 999; up to £13 499. These categories were chosen as they separated the data into equal quarters each containing 25% of the sample. Education was coded into five categories: university education, A-level and equivalent, GCSE/vocational, other/still studying, and none. Working status was coded into two categories: full-time job versus no full-time job; as was housing tenure: owner occupied (owned outright or bought with a mortgage) versus other. These thresholds are based on previous research.^9,21–23^

A composite score was also derived to evaluate how far this added predictive value over any one of the individual SEP measures.^10,24^ The derived composite score was found to have moderate internal consistency (standardized Cronbach’s alpha of 0.6).^25^ This composite score was derived using Multiple Correspondence Analysis in the FactoMineR package.^26^ Weights for the composite score comprised of those for the first three components identified in the Multiple Correspondence Analysis; the assumption being that the variation explained by these is sufficient to adequately represent the original values.^27^ Unity-based normalization was used to allow easier comparison with the dummy variables (ie, it had a range of 0–1).

#### Covariates

Data were also collected on participants’ age, gender, and ethnicity.

### Analysis

The analysis plan was preregistered on the Open Science Framework (https://osf.io/cq8ua).

All analyses were conducted in R Studio. Prevalence of smoking was weighted using a rim (marginal) weighting technique. Missing data were imputed by multiple imputation using the Amelia 11 package.^28^ The number of imputed data sets were based on previous recommendations (ie, *n* = 20)^29^ and results combined using Rubin’s Rules.^30^ All the independent variables including the six indicators of SEP, gender, age, and ethnicity were used in the imputation models.

The extent of missing data was as follows: *n* = 544 (0.45%) for ethnicity, *n* = 717 (0.56%) for car ownership, *n* = 1191 (0.99%) for home ownership, *n* = 421 (0.35%) for employment status, *n* = 48 480 (40.23%) for income, and *n* = 650 (0.54%) for employment status. For all variables, data appeared to be missing at random or missing not at random as missingness depended on age, gender, and ethnicity. Multiple imputation procedures generally rely on the missing at random assumption, but the method can also handle data missing not at random.^31^

#### Associations Between Individual SEP Measures and Smoking Status

Separate generalized linear models were run to assess each association separately between smoking and each SEP measure. The binomial distribution family was specified for each generalized linear model. Each model is reported unadjusted and adjusted for age, gender, and ethnicity. Model fit for each generalized linear model was compared using the (1) adjusted McFadden pseudo *R*-squared, (2) Akaike information criterion (AIC), (3) Bayesian information criterion (BIC), and (4) mean-square deviation (MSE) from 10-fold cross-validation.^32^ Generally, higher *R*-squared values and lower BIC, AIC, and MSE values indicate a better model fit.

As there was a large amount of missing data for income, a sensitivity analysis was also run with complete cases for income. Model fit indices are not given for the complete case sensitivity analysis for income as the AIC, BIC, and MSE all depend on sample size.

#### Multivariable Associations Between SEP Measures and Smoking Status

The predictive ability of each SEP variable when adjusting for all others was assessed with ridge regression due to the high levels of multicollinearity. Ridge regression works by shrinking coefficients, with unimportant terms driven towards zero. The degree of penalization, λ, is known as the ridge factor and must be estimated prior to data analysis. To choose λ, a cross-validation approach was used whereby various models were fitted to the training set with different values of λ. The model was selected which gave the simplest regularized model (where the cross-validated error was within one standard error of the model with minimum λ). Ridge regression leads to coefficients that are slightly biased downwards but with the trade-off of much smaller standard errors and therefore large improvement in the precision of regression coefficients.^5,33,34^

## Results

Between March 2013 and January 2019, data were collected from 120 496 adults aged 16+. Of these, 18.04% (95% confidence interval 17.52 to 18.55, *n* = 21 720) were smokers. Descriptive statistics for the sample are given in Table 1.

**Table 1.** Descriptive Statistics

	Smokers (*n* = 21 720)		Nonsmokers (*n* = 98 776)	
	%	*n*	%	*n*
Age				
16–24	19.25	4164	14.53	14 369
25–34	20.07	4343	13.43	13 280
35–44	16.28	3523	13.86	13 698
45–54	17.02	3682	14.50	14 337
55–64	14.32	3098	15.71	15 534
65+	13.06	2827	27.96	27 641
Female	47.05	10 180	49.85	49 283
White ethnicity	89.25	19 312	83.59	82 631

### Associations Between Individual SEP Measures and Smoking Status

Table 2 shows the results of the generalized linear models assessing the association between each SEP measure and smoking status adjusted for age, gender, and ethnicity (and Supplementary Table S1 reports the unadjusted results). Table 3 and Supplementary Table S2 give the fit indices and MSE from the 10-fold cross-validation for the models reported in Table 2 and Supplementary Table S1, respectively. All SEP measures were significantly associated with smoking status in the analysis adjusted for age, gender, and ethnicity. Comparison of the AIC, BIC, *R*-squared, and MSE from the cross-validation suggested that the strongest predictor was the composite score followed closely by housing tenure, educational qualifications, and social grade.

**Table 2.** Results of the Adjusted Logistic Regressions (for Gender, Age, and Ethnicity) Assessing the Association Between Individual Measures of SEP and Smoking

	Smoker % (*n*)		Nonsmoker % (*n*)		OR_adjusted_	95% CI	*p*
Tenure							
Owns home	11.20	(8333)	88.80	(66 068)	1	1	1
Does not own home	28.87	(13 306)	71.13	(32 789)	3.12	3.01 to 3.22	<.001
Employment							
In full-time work	17.87	(10 041)	82.13	(46 146)	1		
Not in full-time work	18.03	(11 598)	81.97	(52 711)	1.68	1.62 to 1.74	<.001
Income							
Quartile 1 (£50 000+)	12.43	(3043)	87.57	(21 436)	1		
Quartile 2 (£25 000–49 999)	16.15	(5756)	83.85	(29 872)	1.48	1.41 to 1.56	<.001
Quartile 3 (£13 500–24 999)	19.55	(4623)	8045	(19 024)	2.11	2.00 to 2.22	<.001
Quartile 4 (up to £13 499)	22.27	(8219)	77.63	(28 525)	2.62	2.50 to 2.74	<.001
Income without multiple imputation							
Quartile 1 (£50 000+)	10.70	(1591)	89.30	(12 276)	1		
Quartile 2 (£25 000–49 999)	16.12	(3596)	83.88	(19 712)	1.47	1.36 to 1.56	<.001
Quartile 3 (£13 500–24 999)	20.83	(3256)	79.17	(12 374)	1.88	1.75 to 2.01	<.001
Quartile 4 (up to £13 499)	26.23	(5036)	73.73	(14 137)	2.18	2.03 to 2.33	<.001
Education							
University	10.56	(3703)	89.44	(31 370)	1		
A level and equivalent	18.42	(4158)	81.58	(18 417)	1.76	1.68 to 1.85	<.001
GCSE/vocational	23.13	(7846)	76.87	(26 070)	2.65	2.54 to 2.78	<.001
Other/still studying	16.12	(1584)	83.88	(8241)	1.99	1.86 to 2.12	<.001
No post-16 qualifications	22.76	(4349)	77.24	(14 757)	4.00	3.80 to 4.21	<.001
Car ownership							
Owns car	15.74	(3742)	84.26	(20 036)	1		
Does not own car	18.50	(17 897)	81.50	(78 821)	1.25	1.21 to 1.31	<.001
Social grade							
AB	9.06	(2575)	90.94	(25 835)	1		
C1	15.25	(5874)	84.75	(32 657)	1.74	1.66 to 1.83	<.001
C2	20.96	(5033)	79.04	(18 982)	2.63	2.50 to 2.77	<.001
D	24.81	(4267)	75.19	(12 931)	3.42	3.24 to 3.61	<.001
E	31.52	(3890)	68.48	(8453)	5.26	4.97 to 5.57	<.001
	*M*	SD	*M*	SD	OR_adjusted_	95% CI	*p*
Composite	0.52	0.25	0.40	0.25	1.83	1.80 to 1.86	<.001

Standardized coefficients are given for the composite score; separate models were run for each SEP measure. CI = confidence interval; GCSE = General Certificate of Secondary Education; OR = odds ratio; SD: standard deviation; SEP = socioeconomic position.

**Table 3.** Model Fit Statistics (*R*-Squared, AIC, and BIC) and Mean-Squared Prediction Error From 10-Fold Cross-Validation for the Regression Models Presented in Table 2

SEP predictor variable	*R* ^2^,*100	AIC/BIC	MSE
Tenure	7.600	104 846.5/104 933.8	0.13614
Employment	4.278	108 614.9/108 702.2	0.14119
Income	5.211	107 560.0/107 666.7	0.13994
Education	6.533	106 062.9/106 179.3	0.13776
Car ownership	3.578	109 409.5/109 496.8	0.14248
Social grade	7.270	105 226.3/105 342.7	0.13655
Composite	8.517	103 808.3/103 893.6	0.13455

MSE = mean-square deviation; SEP = socioeconomic position.

### Multivariable Associations Between SEP Measures and Smoking Status

Table 4 reports the results from the selected ridge regression model adjusted for age, gender, and ethnicity. Supplementary Figures S1 and S2 describe the ridge regression models at different values of λ. Based on coefficient estimates (odds ratio), the strongest predictor of smoking status was housing tenure. Social grade, educational qualifications, and income were also good predictors of smoking status. Findings were similar for the best ridge models adjusted for all measures of SEP but with no adjustment for age, gender, and ethnicity (Supplementary Table S3).

**Table 4.** Results of the Ridge Regression at Optimal Values of Lambda (Adjusted for Age, Gender, and Ethnicity, and All Measures of SEP)

	OR	95% CI
Tenure		
Owns home	1	
Does not own home	2.01*	2.00 to 2.02
Employment		
In full-time work	1	
Not in full-time work	1.03*	1.03 to 1.04
Income		
Quartile 1 (£50 000+)	1	
Quartile 2 (£25 000–49 999)	1.06*	1.05 to 1.07
Quartile 3 (£13 500–24 999)	1.18*	1.17 to 1.19
Quartile 4 (up to £13 499)	1.23*	1.22 to 1.23
Education		
University	1	
A level and equivalent	1.15*	1.14 to 1.16
GCSE/vocational	1.48*	1.46 to 1.49
Other/still studying	1.12*	1.11 to 1.14
No post-16 qualifications	1.48*	1.48 to 1.51
Car ownership		
Owns car	1	1
Does not own car	1.05*	1.05 to 1.06
Social grade		
AB	1	
C1	1.04*	1.04 to 1.05
C2	1.29*	1.28 to 1.30
D	1.39*	1.38 to 1.40
E	1.78*	1.76 to 1.79

Standard errors for ridge regression are biased to allow accurate estimation of coefficients; CI = confidence interval; GCSE = General Certificate of Secondary Education; OR = odds ratio; SEP = socioeconomic position. *Significant at p < .05.

## Discussion

### Summary of the Findings

In the ridge regression analysis, the selected model including all SEP measures indicated that the strongest individual predictor of smoking status was housing tenure, followed by educational qualifications, social grade, and income. These findings were supported by indices of model fit and 10-fold cross-validation from separate models including only single measures of SEP and adjusted for age, gender, and ethnicity.

### Comparison to Previous Studies

Previous studies have found housing tenure to be strongly related with smoking.^17^ In 2016, 33% of those who lived in social housing were cigarette smokers compared with approximately 10% of those who owned their home.^35^ There are several possible explanations for this, including that housing tenure captures aspects of the local environment and other aspects of “owned” homes relative to rented and social housing.^7^ Those in social housing also often experience greater levels of depression and poor mental health which themselves are also associated with smoking behavior.^36^

Educational qualifications also emerged as a good independent predictor of smoking status. Previous studies have reported that higher educational attainment is associated with smoking behavior.^37,38^ It is possible that education increases individuals’ uptake of information on the health implications of smoking, that they are simply more likely to be exposed to such information, or that those with higher educational qualifications are more likely to use their resources on maintaining their health. It may also be that there is no causal association but that future-orientated individuals invest more in their health and are also more educated.^39^

Social grade is a classification system based on occupation. Over the past several decades, those working in manual occupations have consistently been identified as the highest-risk group for smoking.^40^ As social grade is more closely connected with working conditions than the other SEP indicators, work-related factors present in manual labor may contribute to this higher risk. These include job stress, hazardous working conditions, place of work, and the meaning of smoking among workers.^41^

Household income also offered some predictive power and gives a good indication of the standard of living and life chances of a household. However, its use may be limited as participants are often reluctant to answer questions regarding finances, as evident by the large amounts of missing data in the current study. Household members may also not have equal access to the income which blurs the association with smoking.^42^ Indeed, previous studies have highlighted the complexity of the association between smoking and income.^43^

Although both car ownership and employment status were predictive of smoking, they did not appear to perform as well when judged against the other measures of SEP. Previously, it had been thought that car ownership was a good indicator of affluence due to the costs associated with purchase and maintenance; however, questions have been raised as to whether it is still an appropriate measure.^7,8^ Employment status may have a weaker association with smoking than other SEP measures as smoking adoption generally occurs in younger groups before employment starts to play a role.^44^ Crucially, adjustment for age, gender, and ethnicity increased the association of employment with smoking status which may be due to its complex relationship with age. Generally, employment status is more of a stable indicator of higher SEP in older age groups. In contrast, employment status at younger ages may in fact be associated with greater likelihood of smoking due to providing financial independence to purchase cigarettes.^45^ Another caveat with using employment measures is that those who are retired may have high levels of disposable income but are routinely grouped with other nonworking groups, such as the long-term unemployed.

Although the majority of studies to date have generally only considered one or two measures of SEP, a handful of studies have made comparisons among multiple measurements. For example, Laaksonen et al.^46^ compared measures of education, occupational status, household income per consumption unit, housing tenure, economic difficulties, and economic satisfaction. Similar to the present study, they also found housing tenure to be a strong predictor of smoking status. However, as they failed to account for multicollinearity among measures, coefficients indicating the strength of association may have been biased.^4^ Hiscock et al.^1^ provide a narrative review of studies looking at the association between smoking status and SEP but did not directly assess the optimal measure. Others have addressed multicollinearity with the use of composite measures.^47–49^

It should be noted that in the current study the composite score outperformed all individual measures in predicting smoking status in the simple regression models. The popularity of composite scores stems from the recognition of the multifaceted nature of SEP but they come with significant costs for use in surveys. In this study, we used a weighted composite measure of SEP, whereas previous studies have often used a summation of the products of variable values and therefore equal weights. The advantage of weighting is that individual measures which are more predictive are given greater influence in the composite score.^50^ However, composite scores also have several limitations as compared with individual measures of SEP. Financial and logistical constraints mean it is not always possible to assess multiple measures of SEP within the same survey and there are statistical challenges in relation to the selection of weights. They can also hide important monotonic relationships.^51^

### Implications

These findings have several implications. First, they provide guidance as to which measures one could use when trying to identifying individuals most at risk from smoking. In effect, this can help to tailor interventions and supports the concept of personalized medicine.^52^ Secondly, although these findings suggest that ideally multiple measures of socioeconomic status should be used in population surveys, they offer some guidance as to which socioeconomic measures to choose when there are financial or logistical constraints and the goal is to assess associations with smoking. Thirdly, local authorities in England have control and responsibility for both social housing and public health, including smoking cessation. These findings support arguments for targeted smoking cessation campaigns and provision of neighborhood services. This would support the 2017 tobacco control plan for England which called for targeted action to address inequalities.^53^ Finally, these findings are also largely consistent with the optimal SEP predictors identified for frequency and quantity of alcohol intake,^12^ and may reflect the overlap in the two behaviors.^13^

### Future Research

Several conceptual models exist which can attempt to explain the links identified here between SEP and smoking and it will be important to assess these in future research. For example, according to the COM-B model variation in smoking prevalence across SEP groups can be explained by differences in capability (ie, an individual’s psychological and physical ability to engage in the activity), opportunity (ie, all the factors that lie outside the individual that make the behavior possible) and motivation (ie, all the brain processes that energize and direct behavior).^54^ Specifically, psychological capabilities such as perceived control appear important and opportunity in the form of social ties and access to stop smoking cessation medication and support.^55,56^ Motivational factors include situational self-efficacy, beliefs and affective states.^57,58^

Previous studies have shown that lower SEP is associated with poorer mental health, a larger proportion of friends and family members who smoke, poorer social support, greater nicotine dependency, and lower self-confidence.^59–62^ These factors are all associated with a greater propensity for smoking.^59,62,63^ Motives to quit also differ as a function of SEP group, with those of lower SEP more likely to state cost and health problems.^64^ Motivation to quit and attempts to stop appear to be similar across groups although success rates differ, being lowest among the most disadvantaged groups.^65^

### Advantages and Limitations

This study has several advantages, including the use of data from a large household survey of adults in England and application of a novel statistical technique, ridge regression, to assess the optimal SEP predictors of smoking status. However, this study also has several limitations which must be considered. Regarding the study design, as with all cross-sectional observational surveys, caution should be taken when assigning cause and effect. It may be the case that SEP has a direct influence on smoking behavior, but smoking behavior may also influence some of the SEP measures. For example, smoking can result in significant disability/chronic morbidities which may limit how much paid work someone is able to do. Also, while the sample was designed to be representative, there is a risk of bias in terms of the characteristics of those who agree to participate. There is also a risk that respondents may fail to report their smoking.

In terms of measurement limitations, although this paper assessed a wide range of SEP measures it did not address the social capital aspect of SEP, which reflects the networks of relationships among people who live and work in a particular society. This is something which may require further consideration, as family and friend networks are associated with health outcomes.^66^ We also used smoking status instead of a measure of smoking intensity such as pack-years, and classified nondaily smokers as smokers. While intensity of smoking is important as some smoking-related illnesses do exhibit a linear dose–response relationship, recent research suggests most of the cardiovascular risk from smoking comes from the first few cigarettes a day,^67^ therefore smoking status is an important health indicator.

Regarding the analysis, although we adjusted for several demographic characteristics, some of these findings may be accounted for by other factors which are correlated with SEP, including area level deprivation. In addition, a number of the variables in this study were categorized after data collection (eg, employment status), and it is conceivable that this may preclude some associations. Finally, the meaning of different SEP measures—especially those related to occupation and education—can change with age. We included age as an additional covariate in an attempt to mitigate against this.

Lastly, in terms of generalizability, findings may not be applicable to other countries with very different socioeconomic compositions and tobacco control policies. There are also likely to be geographical differences within England. For example, car ownership may be a stronger indicator outside of cities with major public transport links.^68^ This study also only assessed how SEP measures are associated with smoking but not why they are. Additional qualitative and longitudinal research is needed to address this question. Part of the explanation relates to how the SEP measures assess somewhat different (albeit related) constructs, rather than simply being better or worse assessments of SEP.

In conclusion, of all the socioeconomic measures, housing tenure appears to be the strongest independent predictor of smoking in England, followed by social grade, educational achievements and income. Employment status and car ownership have the lowest predictive power.

## Supplementary Material

A Contributorship Form detailing each author’s specific involvement with this content, as well as any supplementary data, are available online at https://academic.oup.com/ntr.

ntaa030_suppl_Supplementary_information

ntaa030_suppl_Supplementary_Taxonomy_Form

## Data Sharing

For access to the data please contact the lead author or Dr Jamie Brown (Jamie.Brown@ucl.ac.uk).

Additional references can be found in the supplementary file.
