# Correction to: Independent Associations Between Different Measures of Socioeconomic Position and Smoking Status: A Cross-Sectional Study of Adults in England

**DOI:** 10.1093/ntr/ntae098

**Published:** 2024-05-02

This is a correction to: Emma Beard, Jamie Brown, Sarah E Jackson, Robert West, Loren Kock, Sadie Boniface, Lion Shahab, Independent Associations Between Different Measures of Socioeconomic Position and Smoking Status: A Cross-Sectional Study of Adults in England, Nicotine & Tobacco Research, Volume 23, Issue 1, January 2021, Pages 107–114, https://doi.org/10.1093/ntr/ntaa030

Data were not collected from January 2015 on car ownership. This was not identified in the previous analysis as the missing data had been coded as ‘0’ rather than ‘NA’. In the published manuscript it is reported that 0.56% of the data were missing for car ownership when in fact 70% of the data were missing. The analysis was re-run with multiple imputation. This did not substantially change the findings, with housing tenure remaining the strongest predictor followed by social grade, educational qualifications and income.

*Change to Table 2:* 47.41% (n=10258) of smokers did not own their own car. 35.81% (35402) of non-smokers did not own their own car. Smokers had 2.95 higher odds of not owning their own car (95%CI 2.74 to 3.17, P<0.001).

*Change to Table 3:* The fit indices for car ownership are R^2^*100 4.220, AIC/BIC 108677.0/108764.3, MSE 0.14168

*Change to Table 4:* In the ridge regression analysis, an OR of 1.18 was found for car ownership. See the full changes in Table 1 below.

The analysis was also repeated removing car ownership. Again, this did not substantially change the findings. Full amendments for both analyses can be found on the OSF: https://osf.io/bgf3q/. These details have been corrected only in this correction notice to preserve the published version of record.

**Table 1.** Results of the ridge regression at optimal values of lambda (adjusted for age, gender and ethnicity, and all measures of SEP)

	OR	95%CI
***Tenure***		
Owns home	1	
Does not own home	1.97*	1.97 to 1.98
***Employment***		
In full time work	1	
Not in full time work	1.02*	1.02 to 1.03
***Income***		
Quartile 1 (£50,000+)	1	
Quartile 2 (£25,000 to £49,999)	1.01*	1.00 to 1.01
Quartile 3 (£13,500 to £24,999)	1.10*	1.09 to 1.10
Quartile 4 (up to £13,499)	1.16*	1.16 to 1.17
***Education***		
University	1	
A level and equivalent	1.16*	1.15 to 1.16
GCSE/vocational	1.49*	1.48 to 1.49
Other/still studying	1.13*	1.12 to 1.14
No post 16 qual	1.50*	1.49 to 1.50
***Car ownership***		
Owns car	1	
Does not own car	1.18*	1.18 to 1.18
***Social-grade***		
AB	1	
C1	1.06*	1.05 to 1.06
C2	1.31*	1.31 to 1.32
D	1.40*	1.39 to 1.41
E	1.73*	1.73 to 1.74

**Note:** standard errors for ridge regression are biased to allow accurate estimation of coefficients; significant at p<0.05

