# Supplementary material for: Independent Associations Between Different Measures of Socioeconomic Position and Smoking Status: A Cross-Sectional Study of Adults in England
Source: Nicotine Tob Res. 2020 Feb 6;23(1):107–14. doi: 10.1093/ntr/ntaa030 (PMC7789954; doi:10.1093/ntr/ntaa030)
Supplement: ntaa030_suppl_Supplementary_information [file ntaa030_suppl_supplementary_information.docx]

**Supplementary Table 1:** Results of the unadjusted linear regressions assessing the association between individual measures of socio-economic status and smoking status

|  | OR | 95%CI | P |
| --- | --- | --- | --- |
| **Tenure**  Owns home  Does not own home | 1  3.22 | 3.12 to 3.32 | <0.001 |
| **Employment**  In full time work  Not in full time work | 1  1.01 | 0.98 to 1.04 | 0.463 |
| **Income**  Quartile 1 (£50,000+)  Quartile 2 (£25,000 to £49,999)  Quartile 3 (£13,500 to £24,999)  Quartile 4 (up to £13,499) | 1  1.36  1.71  2.03 | 1.30 to 1.42  1.63 to 1.80  1.94 to 2.12 | <0.001  <0.001  <0.001 |
| ***Income without multiple imputation*** % (n) |  |  |  |
| Quartile 1 (£50,000+) | 1 |  |  |
| Quartile 2 (£25,000 to £49,999) | 1.60 | 1.51 to 1.71 | <0.001 |
| Quartile 3 (£13,500 to £24,999) | 2.20 | 2.06 to 2.34 | <0.001 |
| Quartile 4 (up to £13,499) | 2.97 | 2.80 to 3.16 | <0.001 |
| **Education**  University  A level & equivalent  GCSE/vocational  Other/still studying  No post 16 qual | 1  1.91  2.55  1.63  2.50 | 1.82 to 2.01  2.44 to 2.66  1.53 to 1.73  2.38 to 2.62 | <0.001  <0.001  <0.001  <0.001 |
| **Car ownership**  Owns car  Does not own car | 1  1.22 | 1.17 to 1.26 | <0.001 |
| **Social-grade**  AB (highest)  C1  C2  D  E (lowest) | 1  1.81  2.66  3.31  4.62 | 1.72 to 1.90  2.53 to 2.80  3.14 to 3.49  4.37 to 4.88 | <0.001  <0.001  <0.001  <0.001 |
| **Composite** | 1.60 | 1.57 to 1.62 | <0.001 |

**Note:**

**Supplementary Table 2:** Model fit statistics (R-squared, AIC and BIC) and mean squared prediction error from 10-fold cross validation for the regression models presented in supplementary Table 1

|  | Unadjusted model | | |
| --- | --- | --- | --- |
| SEP predictor variable | R^2^*100 | AIC/BIC | MSE |
| Tenure | 5.170 | 107588.9/107608.3 | 0.13997 |
| Employment | <0.001 | 113453.8/113473.2 | 0.14734 |
| Income | 1.001 | 112322.4/112361.2 | 0.14598 |
| Education | 2.088 | 111091.8/111140.3 | 0.14460 |
| Car ownership | 0.090 | 113352.7/113372.1 | 0.14722 |
| Social grade | 3.495 | 109495.7/109544.2 | 0.14251 |
| Composite | 3.432 | 109560.6/109580.0 | 0.14268 |

**Supplementary Table 3:** Results of the ridge regression at optimal values of lambda (unadjusted for sex, age and ethnicity)

|  |  | |
| --- | --- | --- |
|  | OR | 95%CI |
| ***Tenure***  Owns home  Does not own home | 1  2.10* | 2.09 to 2.11 |
| ***Employment***  In full time work  Not in full time work | 1  0.87* | 0.86 to 0.87 |
| ***Income***  Quartile 1 (£50,000+)  Quartile 2 (£25,000 to £49,999)  Quartile 3 (£13,500 to £24,999)  Quartile 4 (up to £13,499) | 1  1.04*  1.12*  1.15* | 1.04 to 1.05  1.11 to 1.12  1.14 to 1.16 |
| ***Education***  University  A level and equivalent  GCSE/vocational  Other/still studying  No post 16 qual | 1  1.16*  1.45*  1.05*  1.28* | 1.15 to 1.16  1.44 to 1.46  1.04 to 1.06  1.27 to 1.29 |
| ***Car ownership***  Owns car  Does not own car | 1  1.03* | 1.03 to 1.04 |
| ***Social-grade***  AB  C1  C2  D  E | 1  1.02*  1.27*  1.36*  1.75* | 1.02 to 1.03  1.26 to 1.28  1.35 to 1.37  1.74 to 1.77 |

**Note:** standard errors for ridge regression are biased to allow accurate estimation of coefficients; * significant at p<0.05

**Supplementary File 1:** Choosing the ridge regression model

The results of the unadjusted ridge regression for predicting smoking status at different values of Lambda are given in supplementary Figure 1. Figures 2 show the same results for the model adjusting for age, gender and ethnicity. Moving a short distance from the coefficients for the standard regression (x=0), leads to a rapid decrease in absolute values of the coefficients. Thus, it is clear from this that the standard regression coefficients were overestimating and were unstable.

The optimal Lambda, when the coefficients stabilised, was 0.052 for the unadjusted and 0.040 for the adjusted model.


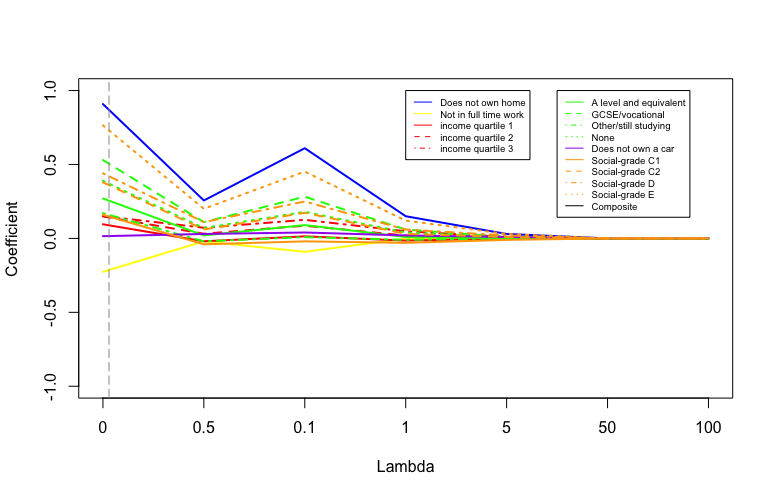


**Supplementary Figure 1:** Results of the ridge regression at different values of Log(Lambda) for predicting smoking (unadjusted)

Note: grey dash line shows point of coefficient stabilisation


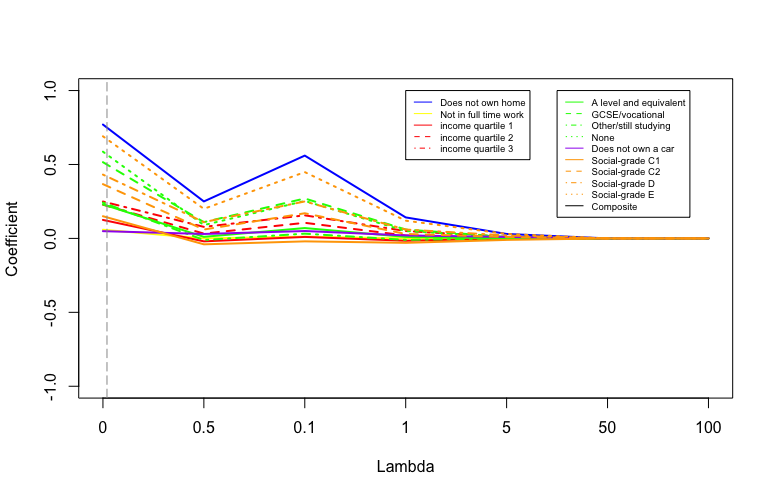


**Supplementary Figure 2:** Results of the ridge regression at different values of Log(Lambda) for predicting smoking (adjusted)

Note: grey dash line shows point of coefficient stabilisation

**References**

51. Ezzrari A, Verme P. A multiple correspondence analysis approach to the measurement of multidimensional poverty in Morocco, 2001–2007: The World Bank; 2012.

52. Barclay M, Dixon-Woods M, Lyratzopoulos G. The problem with composite indicators. BMJ Quality &amp; Safety. 2019;28(4):338-44.

53. Vogenberg FR, Barash CI, Pursel M. Personalized medicine: part 1: evolution and development into theranostics. P & T. 2010;35(10):560.

54. Department of Health. Towards a Smokefree Generation: A Tobacco Control Plan for England. 2017.

55. Michie S, Van Stralen MM, West R. The behaviour change wheel: a new method for characterising and designing behaviour change interventions. Implementation science. 2011;6(1):42.

56. Harwood GA, Salsberry P, Ferketich AK, Wewers ME. Cigarette smoking, socioeconomic status, and psychosocial factors: examining a conceptual framework. Public Health Nursing. 2007;24(4):361-71.

57. Honjo K, Tsutsumi A, Kawachi I, Kawakami N. What accounts for the relationship between social class and smoking cessation? Results of a path analysis. Social science & medicine. 2006;62(2):317-28.

58. Manfredi C, Cho YI, Crittenden KS, Dolecek TA. A path model of smoking cessation in women smokers of low socio-economic status. Health Education Research. 2006;22(5):747-56.

59. West R, Brown J. Theory of addiction: John Wiley & Sons; 2013.

60. Hiscock R, Judge K, Bauld L. Social inequalities in quitting smoking: what factors mediate the relationship between socioeconomic position and smoking cessation? Journal of public health (Oxford, England). 2011;33(1):39-47.

61. Siahpush M, McNeill A, Borland R, Fong GT. Socioeconomic variations in nicotine dependence, self-efficacy, and intention to quit across four countries: findings from the International Tobacco Control (ITC) Four Country Survey. Tobacco Control. 2006;15(suppl 3):iii71-iii5.

62. Tsourtos G, O'Dwyer L. Stress, stress management, smoking prevalence and quit rates in a disadvantaged area: has anything changed? Health Promotion Journal of Australia. 2008;19(1):40-4.

63. Turner LR, Mermelstein R, Hitsman B, Warnecke RB. Social support as a moderator of the relationship between recent history of depression and smoking cessation among lower-educated women. Nicotine & Tobacco Research. 2008;10(1):201-12.

64. Hover SJ, Gaffney LR. Factors associated with smoking behavior in adolescent girls. Addictive behaviors. 1988;13(2):139-45.

65. Pisinger C, Aadahl M, Toft U, Jorgensen T. Motives to quit smoking and reasons to relapse differ by socioeconomic status. Preventive medicine. 2011;52(1):48-52.

66. Kotz D, West R. Explaining the social gradient in smoking cessation: it's not in the trying, but in the succeeding. Tob Control. 2009;18(1):43-6.

67. Berkman LF, Glass T. Social integration, social networks, social support, and health2000. 137-73 p.

68. Hackshaw A, Morris JK, Boniface S, Tang J-L, Milenković D. Low cigarette consumption and risk of coronary heart disease and stroke: meta-analysis of 141 cohort studies in 55 study reports. BMJ. 2018;360:j5855.

69. Beard E, Brown J, West R, Angus C, Kaner E, Michie S. Healthier central England or North–South divide? Analysis of national survey data on smoking and high-risk drinking. BMJ open. 2017;7(3):e014210.
